# Sport fans’ self-esteem and supporting the team that won over the favorite

**DOI:** 10.3389/fspor.2026.1810401

**Published:** 2026-06-03

**Authors:** Endzhe Takemura, Hirotaka Matsuoka

**Affiliations:** 1 Graduate School of Sport Sciences, Waseda University, Tokyo, Japan; 2 Faculty of Business Administration, Reitaku University, Chiba, Japan; 3 Faculty of Sport Sciences, Waseda University, Tokyo, Japan

**Keywords:** feeling-as-information theory, social identification theory, sport fans, team identification, tournament

## Abstract

**Research purpose:**

Most often, when their favorite team loses, sports fans are disappointed by the outcome and hostile to the winning team. However, in the tournaments, they also sometimes voice their support for the team that eliminated their favorite. The present study investigates these phenomena and proposes that fans supporting the team that eliminated the favorite in the tournament is a coping strategy. It is used to help fans mitigate the identity threat when direct competition between the team becomes unavailable. Therefore, the purpose of the present study is to investigate the psychological mechanisms underlying fans’ support for the team that defeated the favorite, from the perspectives of social identification and feelings-as-information theories.

**Research method:**

The questionnaire was distributed via an online panel company to Japanese professional baseball fans. The items included team identification, collective self-esteem, and negative emotions. The team's loss was manipulated using the scenario. PLS-SEM analysis was conducted in the R software.

**Results and findings:**

The relationship between team identification, self-esteem, and supporting the team that won was significant; however, there was no support for the proposition that supporting the team that won acts as a coping strategy. The path from team identification to self-esteem was positive, indicating that higher identified fans had higher self-esteem even after the team lost. Furthermore, the path between self-esteem and supporting the team that won was also positive. Perhaps fans extended their support to promote their division. Contrary to expectations, the negative emotions did not mediate the relationship between team identification and self-esteem.

## 1 Introduction

Team identification (TI) is a core concept in sport management that captures the psychological connection between fans and their teams. Fans who are high in TI experience the team's wins as their own, which leads to positive emotions and improved self-esteem (1). However, if that is true for the wins, the opposite processes are likely to occur after a loss as well. While wins represent success for the associated group (fans), research suggests that sports fans view losses as failures of the team. Faced with the failure of the associated group, the fans react accordingly, with a surge of negative emotions, a drop in self-esteem, and the use of coping strategies to help shield their social selves from the reflected failure.

Coping strategies vary in how fans respond to identity threats. Perhaps the most well-researched coping strategy is CORFing (or cutting off reflected failure), which involves fans temporarily separating their identity from the team (2). The fans might remember past successes or focus on future successes to face failure (3). The strategies mentioned so far focus on the relationship between the fan and the favorite team. Additionally, fans can moderate their relationship with the team that beat their favorite to restore balance. For instance, one prominent example of such a strategy is blasting, or talking badly about the opponent (4). Moreover, sports fans exhibit schadenfreude, or malicious pleasure, when their disliked team loses (5).

The selection of coping strategies appears to be influenced by TI. The fans who are low in TI tend to use CORFing with greater ease than the high-TI fans, whose identities intertwine more strongly with the TI. So, high-TI fans tend to use other coping strategies to maintain their association with a team.

From a managerial point of view, it is beneficial for the team to have high-TI fans, as their loyalty is often reflected in their actions towards the team (e.g., attendance). However, high TI could be associated with undesirable behavior, such as blasting or schadenfreude, especially after the team's loss. Furthermore, problems of hooliganism and sport-related aggression are often highlighted by the press and the general public (for instance, the NHL Stanley Cup riots, football hooliganism, etc.). If high TI fans react to their team's losses with out-group aggression, that is problematic for the team.

However, aggression is not the only reaction to the outgroup, even though it is the most researched. Grieve et al. (6) note that, in tournaments, fans whose favorite team was eliminated were not opposed to supporting a different team, including the team that eliminated them (associated with a high level of basketball fandom). Latypova and Matsuoka (7) reported that some fans of Japanese high school baseball expressed their intention to support the school that beat the favorite. Moreover, Takemura et al. (8) reported that nearly 40% of respondents wished the team that eliminated their favorite would continue winning. In some circumstances, fans show support for the team that beat the favorite (hereafter, STW) rather than being aggressive towards it.The research is still to explain what are the circumstances that trigger that behavior. It could be suggested that STW serves as a coping mechanism, allowing fans to find relief in the idea that at least their favorite team has lost to a strong opponent. Elimination from the tournament results in the absence of direct competition, which impacts the coping strategy. A similar sentiment among fans was noted by Havard (9), who observed that fans were willing to root for their team's rival under the condition that the rival's win would improve the prestige of the favorite team in the absence of direct competition.

Therefore, the present study defines STW as a fan behavior characterized by the fans temporarily extending their support to the team that defeated the favorite. While the evidence in the previous research is fragmented, it could be assumed that the switch is only temporary, so the feelings for the favorite team remain unchallenged. Rather, the loss of the favorite team allows fans to briefly include the winning team in their perception of the ingroup when direct competition between the two teams is unavailable. The present study tests the assumption that STW acts as a coping strategy. While the STW has been noted by the research, a detailed analysis of the underlying factors is yet to be conducted. Understanding the antecedents of STW might shed light on the fans’ coping strategies. Moreover, from a managerial point of view, understanding STW might provide insights into how to appeal to fans whose team lost and help them transition to more peaceful ways to cope with the loss. As for the league, understanding how to help fans find another team to root for may be crucial for the tournament viewer retention.

Thus, the purpose of the present study is to investigate the psychological mechanisms underlying fans’ support for the team that defeated the favorite. More specifically, the present study is designed from the perspectives of social identification theory and feelings-as-information theory.

## 2 Literature review

### 2.1 Social identification theory

Team identification (hereafter, TI) is a powerful feeling that allows fans to share their favorite team's experience as their own. Even though they are not on the field, fans vicariously partake in both the joy of winning and the sorrow of losing. Even more importantly, social identification theory, which underpins the study of team identification, suggests that the team's game outcome (win or loss) influences fans’ self-esteem. Associating with the winning team lifts the self, and losses threaten the self.

In fact, wanting to associate with a well-performing team was identified as one of the main motivations for the individual to become a fan (10). After forming the association, the favorite team's wins can affect how fans view themselves (11, 12). Finally, from the behavioral perspective, sports fans are known to bask in reflected glory (hereafter, BIRGing; 2, 13–15). After the university team's victory, students wore more university apparel to class and used the pronoun “we” to describe the team's result (13). Even two days after the victory, fans’ self-esteem was heightened (1). To sum up, associating with the winning team helps the fans to feel better about themselves.

On the other hand, loss is negatively impacting fans. After the loss, fans estimated their future performance lower than after the win (11) and experienced a drop in self-esteem (16). Fans, however, can use coping to protect the threatened self. For instance, when the defeated team's spectators were provided the opportunity to distance themselves from the team (thus allowing them access to the coping strategy), they reported higher self-esteem as opposed to when they reported their self-esteem first (where coping would be delayed until after the survey; 16). Thus, coping strategies help fans to shield their self-esteem.

Significant research effort has been devoted to describing and categorizing the coping strategies used by sports fans, with cutting off reflected failure, blasting, and boosting (17) being the most prominent. In particular, CORFing (2, 18) or the tendency to temporarily separate the self from the team by, for example, referring to the team as “them” instead of “we”, is often seen as being in the same continuum with BIRGing.

While many factors that influence the choice of coping strategies remain to be determined, one has been shown to be TI. Low-identified fans CORF, but high-identified fans are unable to cut ties with the team, so they are more likely to boost, blast, and focus on a new comparison dimension (NCD; 19).

As for the STW, there is no prior research that allows us to predict which TI type is associated with this coping strategy. However, as it is expected that fans are seeking ways to indirectly increase their favorite team's status rather than simply cutting off the relationship, it is assumed that fans with higher TI will be more likely to use STW, similarly to NCD (19).

Therefore, the hypotheses were formulated as follows:

H1. After exposure to the loss scenario, highly identified fans will experience lower self-esteem.
H2. Fans’ self-esteem will be associated with STW, and fans with low self-esteem will be more likely to engage in STW.

### 2.2 Feelings and feelings-as-information theory

Affect-as-information theory states that moods impact an individual's behavior and judgment, even if the subject is not directly related to the cause of mood, e.g., having a more positive outlook on life on a sunny day (20, 21). Later, feelings and emotions, even though theoretically distinct from moods, were shown to have similar effects.

Then, the game results could be expected to alter the fans’ emotional state. In research, the loss of the favorite team led to increased estimates of the likelihood of war in the Middle East (22), decreased approval ratings for the president, and satisfaction with the university (23). In economic research, game results have been used as a proxy for the moods themselves when working with large amounts of secondary data, e.g., court records (24).

The research on sports fans backs it up. Wins are associated with positive moods, and losses with negative moods (4, 25–27). Interestingly, TI impacted the intensity of the experienced emotions (4). It also served as a factor differentiating among negative emotions (26). After the loss, low-identified fans were more likely to be sad, and high-identified fans were more likely to be angry.

Finally, mood or feelings have been shown in research to mediate the relationship between game result and spectators’ self-esteem (1, 28, 29). When taken together with the findings that feelings mediate the relationship between TI and coping strategies (4, 26), which are thought to be activated because of the self-esteem threat, the present study proposes that feelings will be associated with both TI and self-esteem following the team's loss.

Therefore, the following hypotheses were formulated:

H3: Following the team’s loss, anger will be associated with both TI and self-esteem.
H4: Following the team’s loss, sadness will be associated with both TI and self-esteem.

Moreover, following the Bernache-Assollant et al. (19), the present study included self-conscious emotions in addition to primary emotions. Self-conscious emotions are associated with identity and society's assessment of the self (30), as they develop later in childhood and depend on the presence of others. Self-conscious emotions appraised in response to negative events are guilt and shame. In the context of sports spectatorship, because consumers do not directly contribute to the game, guilt is unlikely to apply. However, shame was positively associated with the coping strategy in the new comparison dimension (19).

Therefore, the final hypothesis was formulated as follows:

H5: Following the team's loss, shame will be associated with both TI and self-esteem.

## 3 Methodology

### 3.1 The context of the present study

Evidence of STW among sports fans comes from studies conducted in tournament settings (6, 7). It could be argued that the elimination structure of tournaments prevents direct competition between the losing and winning teams, forcing fans to resort to STW to improve their favorite team's status. An example of similar fan behavior could be found in Havard's (9) study on rival fans.

Therefore, the present study was built around a tournament. Japan's Nippon Professional Baseball (NPB) League's postseason was selected as a target because NPB is one of the most prominent leagues in Japan, thereby easing data collection. NPB's postseason is organized as a two-stage tournament in which the Pacific and Central league winners are determined in the first stage, and the NPB champion is selected in the final stage. The process of elimination is, therefore, two-fold. First, the six teams are excluded from the playoffs based on their records, and then another four teams are eliminated in the first stage.

While the settings of previous studies resemble the latter situation, the present study focused on the six teams that did not make the playoffs in 2025. That change was made to reduce the impact of the direct emotional involvement of the fans of the four teams eliminated just days before the postseason. As for the outcome variable, because the target teams lost the chance to participate in the tournament due to their lower ranking, the main target of this study was whether fans would support the representative from the same league (Central or Pacific). In the 2025 playoffs, each of the representative was the champion of its’ respective league. Furthermore, the amount of news coverage of the playoffs emphasized the lower placement of the favorite team and create the tension between the favorite team and the league's champion.

### 3.2 Development of the questionnaire and measures

Upon starting the questionnaire, the respondents were asked to indicate their favorite team from the teams not participating in the playoffs and rate their association with this team [Team ID scale, developed by Trail and James (31), and adapted to Japanese by Nakazawa and Yoshida (32)]. Next, the fictitious scenario describing the loss of the target team was presented. The scenario was created to mimic the format of an article from Yahoo News, one of the most popular sources of baseball-related information in Japan. For the scenario, the researchers first drafted it in English following Yahoo News examples, and then used the AI chatbot ChatGPT to translate it into Japanese, with the style and tone specified to match Yahoo News. It was designed to remind the respondents of the game in which their team had lost the chance to participate in the Japan Series (based on the team rankings within the league). One of the authors, who is a native Japanese speaker and regularly uses Yahoo news checked and finalized the scenario.

Two manipulation checks on the article's content were included to ensure respondents read and comprehended the stimulus article. The respondents were asked to choose which team the article described and what the theme of the article was.

Next, respondents’ self-esteem [via the collective self-esteem scale, adapted by Deguchi et al. (33), and originally developed by Luhtanen and Crocker (34)] and the team they would wish to win the Japan Series. The items were formulated based on those used by Grieve et al. (6) and were descriptive in nature. The target item for the dependent variable read as “I wanted (the team) to win, because (the team) is the representative of the league”. For the smaller samples, the usage of single-item measures in SEM was supported in previous research (35).

Finally, because clearly thinking about the emotion may remove its effect on the dependent variable, three negative emotions (anger, sadness, and shame) were assessed at the end. For anger and sadness, the items used by Crisp et al. (26) were adapted from those originally developed by Mackie et al. (36), while the items used by Bernache-Assollant et al. (19) were adapted to assess shame [originally from Johns et al. (37)]. During the adaptation process, the number of items was reduced to accommodate the specifics of the Japanese language and ensure that the wording in the questionnaire would be used by native speakers in relation to sports. Two bilingual graduate students and two bilingual professors specializing in sport management were consulted to ensure that the translation was correct. After the emotion measurement, respondents were thanked and debriefed.

The questionnaire was submitted to the Ethics Review Committee on Research with Human Subjects of the researcher’s home university (application number 2025-443) and was approved on October 15, 2025.

Finally, pretest was conducted using the sample of Japanese undergraduate students enrolled in the faculty of sport sciences in a prominent Japanese university (*n* = 28). The wording was slightly changed based on feedback from the pretest participants.

### 3.3 Data collection

An online questionnaire was distributed through the survey panel company from November 10th to November 12th, 2025, after the Japan Series 2025 final, the final stage of NPB's 2025 postseason playoffs. The fans of the six teams that did not compete in the postseason were targeted for data collection to ensure they were not directly emotionally involved in the Japan Series 2025.

Finally, during the present data collection, the data for the two studies were collected simultaneously and randomly split after cleaning procedures were completed and before analysis. Upon accessing the invitation link, respondents were randomly assigned to one of six conditions. The present study required respondents only from one condition, so more respondents were allocated to the relevant group with the intention of splitting the sample into 2 before analysis.

Appropriate sample sizes were determined using G*Power (powe*r* = 80%, significance criterion = 5%, effect size = 25%). A minimum of 158 samples was suggested for the second study, and for the present study, a minimum of 138 samples; thus, the total sample size of 296 was determined to be sufficient.

### 3.4 Sample characteristics

2,808 responses were collected, of which 868 failed the manipulation check. Furthermore, responses with straight-line answers (*n* = 338) were excluded because they indicated a lack of attention, resulting in a sample of 1,602 responses (578 responses in the target condition), which were then randomly divided into 2 samples. The sample used for the present study consisted of 375 responses.

Further cleaning procedures were conducted. Firstly, respondents who did not have an opinion on the league's representative team (“Neither yes nor no”; *n* = 139) were excluded from the present analysis, leaving a final sample of 236 responses.

71.2% of respondents (*n* = 168) were male and 28.8% were female (*n* = 68). The mean age of the sample was 53.39 years, ranging from 20 to 79. The fans of the Central league of NPB comprised 60.6% of the sample (*n* = 143), and the fans of the Pacific league comprised 39.4% (*n* = 93). The demographic data of the sample are presented in Table 1.

**Table 1:** Demographic characteristics (n = 236).

	Variables	*n*	%
Characteristics	Male	168	71.2
	Female	68	28.8
Age (*Mean* = 53.39)	18–24 years old	2	0.8
	25–34 years old	10	4.2
	35–44 years old	40	16.9
	45–54 years old	66	28.0
	55–64 years old	79	33.5
	65 + years old	39	16.5
League affiliation of the favorite team	Central	143	60.6
	Pacific	93	39.4
STW (Would want to support the team that won)	Yes	70	29.7
	No	166	70.3

## 4 Results

The present study used PLS-SEM as the main analytical tool for two reasons. Firstly, PLS-SEM does not require a large dataset and is not dependent on the data distribution. Secondly, it provides greater freedom in specifying the structural model than other commonly used SEM tools, e.g., allowing the dependent variable to be binary. The analysis was carried out in R using the “SEMinR” package (38), and the guidelines of Hair et al. (39) were followed.

According to the PLS-SEM procedure, firstly, the measurement model was assessed (see Table 2). Factor loadings for all items ranged from .93 to .97, exceeding the recommended threshold of .708. The Cronbach's alphas for all constructs ranged from .882 to .961, indicating that internal consistency was established. Convergent reliability was established using AVE values, which all exceeded 0.5 (0.894–0.927). Finally, rhoC and rhoA values exceeded the recommended threshold; however, values above 0.9 were observed, suggesting data overfitting due to straight-lining. The possible redundancy of the items may undermine reliability; however, the discriminant validity tests were acceptable. Furthermore, the measurements were established in the previous research on sport consumers (32, 33), so it was decided to retain them in full. As the cleaning procedures were completed and no more straight-lining was reliably detectable, it was decided to proceed with the analysis and urge the following research to collect data offline.

**Table 2:** Factor loadings, internal consistency and reliability.

Variables (*n* = 236)	*M*	*SD*	*λ*	*α*	AVE	rhoC	rhoA
TI	3.17	1.90		.949	.907	.967	.950
TI 1	3.20	1.86	.94				
TI 2	3.02	1.92	.95				
TI 3	3.28	1.92	.97				
Anger	32.38	28.04		.910	.917	.957	.924
Anger 1	32.14	28.13	.95				
Anger 2	32.62	27.94	.96				
Sadness	34.78	27.97		.882	.894	.944	.910
Sadness 1	35.93	27.88	.93				
Sadness 2	33.62	28.05	.96				
Shame	38.88	29.47		.917	.922	.960	.942
Shame 1	36.98	29.95	.95				
Shame 2	40.77	28.99	.97				
Self-esteem	3.65	1.80		.961	.927	.974	.963
Self-esteem 1	3.57	1.84	.96				
Self-esteem 2	3.78	1.83	.97				
Self-esteem 3	3.58	1.74	.96				

Discriminant validity was assessed using the Fornell-Larcker criterion and heterotrait-monotrait indices (HTMT). No issues were detected in the FL criterion analysis, as the AVE for each item was higher than the squared correlations (see Table 3). The HTMT values were all below the recommended value of 0.9 (see Table 4), and the discriminant validity was established.

**Table 3:** FL criteria.

	(1)	(2)	(3)	(4)	(5)	(6)
Variables	*0* *.* *95*					
(2) Anger	0.17	*0*.*96*				
(3) Sadness	0.20	0.74	*0*.*95*			
(4) Shame	0.17	0.77	0.71	*0*.*96*		
(5) Self-esteem	0.78	0.08	0.11	0.12	*0*.*96*	
(6) STW	0.22	0.15	0.08	0.18	0.22	*1.00*

The square root of AVE is presented on the diagonal, and lower triangle presents construct correlations.

**Table 4:** HTMT criteria.

	(1)	(2)	(3)	(4)	(5)	(6)
Variables	—					
(2) Anger	0.18	—				
(3) Sadness	0.22	0.83	—			
(4) Shame	0.18	0.85	0.79	—		
(5) Self-esteem	0.82	0.08	0.12	0.13	—	
(6) STW	0.23	0.16	0.08	0.19	0.22	—

Next, the structural model was assessed. First, the VIF values were analyzed to assess potential collinearity. None of the VIF values have exceeded 5 (TI: 1.043; anger: 3.009; sadness: 2.472; shame: 2.786), thus indicating that no critical collinearity issues were detected.

**Figure 1:**
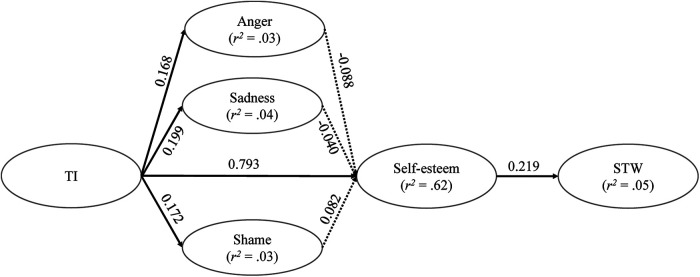
The results of path analysis.

Next, bootstrapping was performed with 10,000 samples. The results are presented in Table 5 and Figure 1. Firstly, it was expected that TI would be negatively related to self-esteem. Although a significant relationship between TI and self-esteem was found, the valence of the relationship was positive [0.793, (0.702; 0.865)], supporting H1 only partially.

**Table 5:** Path coefficients.

	Path coefficient	*t*	95% CI		*f^2^*
			*LL*	*UL*	
Paths	0.168	2.653	0.045	0.292	0.168
TI → Sadness	0.199	2.959	0.075	0.333	0.199
TI → Shame	0.172	2.681	0.053	0.298	0.172
TI → Self-esteem	0.793	18.851	0.702	0.865	0.784
Anger → Self-esteem	−0.088	1.171	−0.234	0.064	0.078
Sadness → Self-esteem	−0.040	0.607	−0.177	0.087	0.110
Shame → Self-esteem	0.082	1.357	−0.037	0.201	0.121
Self-esteem → STW (League's representative)	0.219	3.783	0.105	0.335	0.219

CI, confidence interval; *LL*, lower limit; *UL* , upper limit.

Regarding fans’ self-esteem and STW, the relationship was significant and positive [0.219, (0.105; 0.335)], partially supporting H2. However, the effect size was minimal (r^2^ = 0.048).

As for hypotheses 3 and 5, the results only partially support the proposed relationships. TI and both anger and shame were positively associated. However, no significant association was found between both anger and shame and self-esteem [anger: −0.088, (−0.234; 0.064); shame: 0.082, (−0.037; 0.201)]. As for hypothesis 4, no significant association between sadness and self-esteem was found as well [sadness: −0.040, (−0.177; 0.087)]. Furthermore, the association between TI and sadness was positive, which contradicted the expected direction. Thus, H4 was rejected.

## 5 Discussion

The aim of the present study was to investigate the psychological mechanisms underlying fans’ intention to support the team that eliminated the favorite. The explanation was proposed on the assumption that STW would serve as a coping strategy for fans facing an identity threat from their team's defeat.

The relationships between the TI, self-esteem, and STW were significant; however, the valence was the opposite of the proposed relationship. Results indicate that fans with a high level of TI were more likely to report higher self-esteem, and, in turn, higher self-esteem was associated with STW. It leads to two conclusions regarding self-esteem and STW.

First, the present study used scenarios in which the favorite team was eliminated from the postseason based on the rankings. Because data collection took place after the postseason ended, it was decided to use a more important loss than a regular-season game. However, as the relationship between TI and self-esteem was positive, suggesting that fans did not experience a decrease in self-esteem.

While not directly tested in the present study, one possible explanation could be that the scenario increased the salience of fan identity rather than reminded fans of the team's supposed failure. In this case, the respondents may have responded to the activation of their identity, which would trigger the ingroup bias and lead to the higher estimation of the ingroup, or ingroup favoritism (40). Even when the team loses, fans may be able to derive positive self-esteem from their identification with the team. However, as the present study did not focus on this relationship, further investigation is required in future research.

Furthermore, the present study used a scenario that evoked a memory rather than capitalizing on a real-time event. Further research would benefit from using events that are temporally closer to the data collection point. Fans’ reactions to the loss might change over time, and although the present study suggested that exposure to the loss scenario around the postseason will be sufficient as a stimulus, the possibility that fans had already processed their loss, which affected the results, could not be ruled out.

Furthermore, the postulate that losing leads to a drop in self-esteem among fans is prevalent in the research; however, only a few studies directly measure this relationship. Among those, Knobloch-Westerwick et al. (1) found that fans of the losing team did not experience a decrease in self-esteem following the loss. While surprising, the results of the present study indicate that unidentified factors may be impacting fans’ self-esteem after loss, and that testing the impact of loss on self-esteem will be beneficial for the research. Second, as a higher level of self-esteem was associated with the STW, the present study did not confirm that STW acted as a coping strategy. STW was not caused by a decrease in self-esteem; the fans with higher self-esteem were using it to possibly promote their division and, by extension, their favorite team. Havard (9) has noted a similar behavior among fans, who were supportive of the rival when it helped raise the prestige of the favorite team or conference. The present study has focused on the final stage of the postseason, where the teams representing the sub-leagues of the Japanese professional baseball league meet, so it is possible that the higher levels of identification were especially salient among the baseball fans. As the STW was not associated with low self-esteem, it could be suggested that, rather than a coping strategy, it serves as a way for fans to express their pride in their favorite team.

Another issue to consider is that the explanatory power for the main outcome variable was minimal (r^2^ = 0.048), suggesting that other factors not included in the present research framework are more closely associated with STW, such as personal characteristics.

Regarding the feelings-as-information theory, TI and emotions were positively associated, thus supporting the findings of Bernache-Assollant et al. (4), who confirmed that the fans who associate more strongly with a team are more influenced by the team's results. On the other hand, it was expected that emotions would be negatively associated with self-esteem, as the fans’ perceptions should be altered following the negative affect associated with a loss.

## 6 Limitations and suggestions for the future research

Several limitations should be mentioned regarding the present study.

Firstly, the rhoA and rhoC values exceeded the recommended values, indicating that the model is overfitting the data or that the model fits the data too closely. Even though the data were cleaned using manipulation checks and checked for straight-lining during the later stage of the cleaning process, rhoA and rhoC may indicate straight-lining in the data, which could not be verified through standard data procedures. That might have impacted the reliability of the measurement model. It is advised to implement stricter attention checks when collecting data from panel companies or to use offline data collection.

Next, the present study used a scenario-based approach due to time constraints. It could be suggested that the scenario was not perceived as impactful as the real-life events. The scenario was designed to remind respondents of the loss during the regular season, with the expectation that the absence of the favorite team in the ongoing postseason will magnify fans’ reactions. However, that might not have been sufficient to elicit a fan reaction. Perhaps using an elimination event in real time may be easier for fans to relate to and react to, even though it will require more advanced planning from the researchers. Furthermore, while the present study collected cross-sectional data, the use of longitudinal data collection could be suggested to allow mediation tests.

The present study used a single-item measure to capture fans’ willingness to STW, given the expected small sample size. However, further research might benefit from developing a multi-item measure. It should also be noted that, although only self-proclaimed fans of the target teams were recruited for the study, and respondents who chose a non-target team were not allowed to complete the questionnaire as part of a screening process, the TI mean was only 3.17 on a 7-point Likert scale. Given that the data indicate a higher TI was associated with STW, collecting data from the fan club rather than the general population could help retest the relationships proposed in the present study.

Finally, the sample in the present study had a high mean age (*n* = 53.39) and consisted mainly of male fans, which may have influenced the results. The applicability of the results should be further tested on a younger sample of sports fans.

## 7 Conclusion

The present study has tested the suggestion that supporting the team that won, or the phenomenon of the fans extending their support to the team that eliminated the favorite, is a coping strategy analogous to CORFing, etc. However, the results did not support the suggestion, and the present study did not find a decrease in self-esteem after exposure to stimuli related to the favorite team's loss.

From the theoretical standpoint, that finding contradicts the mainstream research on self-esteem. Several limitations of the present study, such as the use of a fictitious scenario rather than real-life events, might have affected the results; therefore, further testing is needed.

Nevertheless, the present study contributes to the research by providing a closer look at phenomena that have not been in the research spotlight. While about 30% of respondents said they would want to support the team that won, the questions about the reasons behind this behavior remain. It was found to be associated with higher self-esteem driven by stronger team identification; however, the explanatory power was minimal.

While the retesting of the model is needed in further research, it could be suggested that the managers of the teams advancing to the postseason should extend their marketing efforts beyond their teams’ fanbases, as about 30% of the respondents in the present study were willing to temporarily lend their support to the team that won. Highlighting the team as a representative of the league may bring more supporters, even though temporarily, to the stands.

## Data Availability

The raw data supporting the conclusions of this article will be made available by the authors, without undue reservation.
